# Enhancing the energy storage performances of metal–organic frameworks by controlling microstructure†

**DOI:** 10.1039/d2sc03389e

**Published:** 2022-07-18

**Authors:** Jamie W. Gittins, Chloe J. Balhatchet, Simon M. Fairclough, Alexander C. Forse

**Affiliations:** Yusuf Hamied Department of Chemistry, University of Cambridge Lensfield Road Cambridge CB2 1EW UK acf50@cam.ac.uk; Department of Materials Science & Metallurgy, University of Cambridge 27 Charles Babbage Road Cambridge CB3 0FS UK

## Abstract

Metal–organic frameworks (MOFs) are among the most promising materials for next-generation energy storage systems. However, the impact of particle morphology on the energy storage performances of these frameworks is poorly understood. To address this, here we use coordination modulation to synthesise three samples of the conductive MOF Cu_3_(HHTP)_2_ (HHTP = 2,3,6,7,10,11-hexahydroxytriphenylene) with distinct microstructures. Supercapacitors assembled with these samples conclusively demonstrate that sample microstructure and particle morphology have a significant impact on the energy storage performances of MOFs. Samples with ‘flake-like’ particles, with a pore network comprised of many short pores, display superior capacitive performances than samples with either ‘rod-like’ or strongly agglomerated particles. The results of this study provide a target microstructure for conductive MOFs for energy storage applications.

## Abstract 2

The impact of sample microstructure and particle morphology on the energy storage performance of a layered MOF is revealed, with the results providing a target microstructure for MOFs in future energy storage applications.

## Graphical abstract

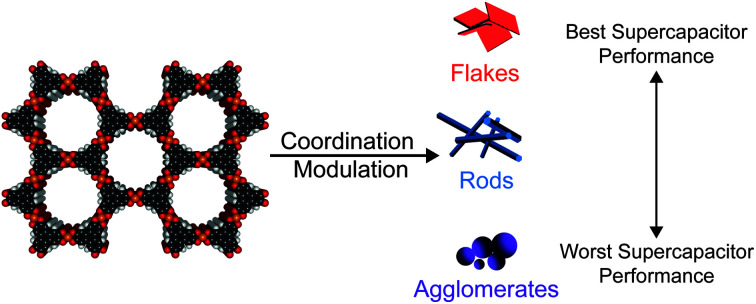

## 1 Introduction

To tackle the climate crisis, a switch to low-carbon technologies, including renewable energy sources and electric vehicles, is urgently required. The development of improved energy storage devices is crucial to facilitate this change.^1^ In recent years, metal–organic frameworks (MOFs), characterised by their combined high intrinsic porosities and electrical conductivities, have emerged as one of the most promising electrode materials for next-generation energy storage devices.^2–10^ Several such MOFs have displayed encouraging performances in supercapacitors with a wide range of electrolytes, exhibiting specific and areal capacitances on par with or exceeding those of state-of-the-art commercial carbon materials.^11–16^ This has raised the prospect of using MOFs in commercial devices, and their well-defined structures make them promising model electrode materials for structure–performance studies.

Many MOFs can be synthesised with a range of different particle morphologies and pore networks, as well as different degrees of particle agglomeration.^17,18^ While significant work has examined the impact of pore structure on the capacitive performances of porous carbon materials and other families of electrically conductive MOFs, little work has been performed to understand how morphology impacts the performances of layered MOFs in supercapacitors.^19–22^ This has hindered the development of layered MOFs for energy storage applications, with potential performance gains to be had from optimising the microstructure. One notable exception is the work of Dincă *et al.*, which studied the influence of sample microstructure on the capacitive performance of Ni_3_(HITP)_2_ (HITP = 2,3,6,7,10,11-hexaiminotriphenylene) in three-electrode cells with 1 M KOH in water electrolyte.^23^ This study suggested that samples with smaller length-to-width aspect ratio particles (‘flatter’ crystals) and weaker particle agglomeration display superior capacitive performances compared to samples with both larger length-to-width aspect ratio particles and greater agglomeration. However, this work primarily relied on batch-to-batch variation to synthesise different samples. This resulted in relatively small differences in observed microstructure, with a variation in the length-to-width aspect ratio over the range of 2.7–12.1, and small changes in agglomeration. Inspired by this study, we were interested in systematically synthesising layered MOFs with a broader range of controlled microstructures, and then using these to comprehensively study the impact of sample microstructure on electrochemical performance.

One way to systematically control the microstructure of MOFs is using coordination modulation.^24,25^ Modulators, which have the same/similar chemical functionality as the organic linker molecule, influence the equilibrium governing the self-assembly process of MOFs by disrupting the formation of coordinative bonds between the linker molecules and the metal ions. Therefore, modulators help to regulate the rate of framework extension and particle growth, thus controlling the final morphology and degree of agglomeration of the resulting materials. Varying the modulator is therefore an efficient way to systematically change the microstructure of MOFs, and can be used to obtain greater differences in microstructure than batch-to-batch variation alone. This method has been successfully employed in previous studies to determine the impact of particle morphology on the physical properties of other MOFs.^26–28^

Drawing on the above, this work uses coordination modulation to synthesise three samples of the layered conductive MOF Cu_3_(HHTP)_2_, (HHTP = 2,3,6,7,10,11-hexahydroxytriphenylene), each with distinctly different microstructures. This approach leads to greater differences in sample microstructure than previous work on this topic, with a variation in the length-to-width aspect ratio over the range of *ca.* 0.01–37. Through this, we present a detailed study into the influence of microstructure on the capacitive performance of Cu_3_(HHTP)_2_ in symmetric supercapacitors with both an organic and ionic liquid electrolyte. These studies conclusively show that sample microstructure has a significant impact on the energy storage performances of layered MOFs. Samples with weakly agglomerated ‘flake-like’ particles, with very small length-to-width aspect ratios (<1), perform best in MOF supercapacitors. These samples, with pore networks comprised of many short pores, display greater rate capabilities with organic electrolytes and significantly higher specific capacitances with ionic liquid electrolytes compared to samples with strongly agglomerated flake crystallites and weakly agglomerated ‘rod-like’ particles.

## 2 Results & discussion

**Fig. 1:**
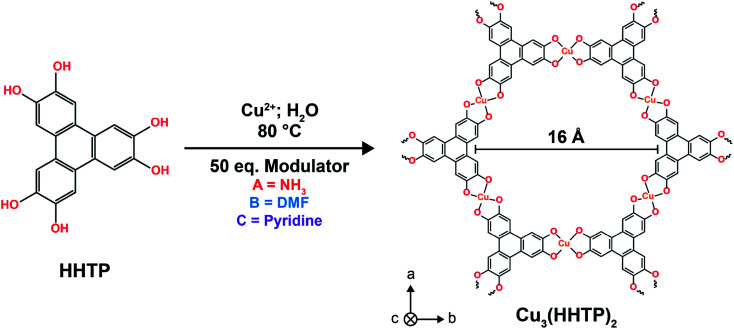
Scheme for the hydrothermal synthesis of Cu_3_(HHTP)_2_ samples from 2,3,6,7,10,11-hexahydroxytriphenylene and a Cu(ii) precursor with different modulators. The structure of Cu_3_(HHTP)_2_ is shown on the left. This MOF is formed of π-d conjugated 2D sheets, which stack to form an extended 3D pore network.

To investigate the influence of microstructure on the energy storage performance of Cu_3_(HHTP)_2_, three samples of Cu_3_(HHTP)_2_ were synthesised with ammonia, dimethylformamide (DMF) and pyridine as modulating agents, respectively (Fig. 1). It has been well studied that varying the identity of the modulator is an effective way to regulate particle morphology and agglomeration, with the different modulators thought to lead to distinct framework growth mechanisms due to differing interactions with the Cu(ii) ions and organic linker molecules in the reaction mixture.^24^ Ammonia, DMF and pyridine were chosen as the modulating agents for this study as previous work indicated that these modulators would produce samples with distinctly different microstructures, including in both particle morphology and the degree of agglomeration.^17,18^ This allowed for a more robust investigation into the influence of microstructure on the capacitive performance of layered MOFs than previously possible. Examining and rationalising the formation mechanisms of these frameworks is beyond the scope of this work.

**Fig. 2:**
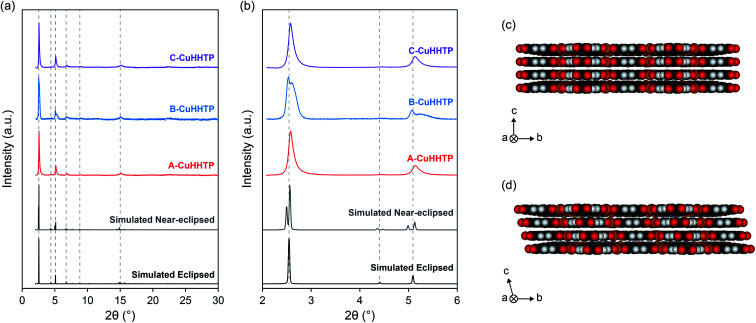
(a) Experimental XRD patterns from A-CuHHTP, B-CuHHTP and C-CuHHTP compared to the simulated XRD patterns from Cu_3_(HHTP)_2_ with both eclipsed and near-eclipsed stacking sequences, and (b) zoomed view to show the differences in peak splitting between the three samples. (c) and (d) Structural models of Cu_3_(HHTP)_2_ with hexagonal eclipsed and monoclinic near-eclipsed crystal structures, respectively.

The formation of Cu_3_(HHTP)_2_ with each modulator was confirmed by powder X-ray diffraction (PXRD) (Fig. 2a and b). The PXRD patterns of ammonia, DMF and pyridine-modulated Cu_3_(HHTP)_2_ (A-CuHHTP, B-CuHHTP, and C-CuHHTP, respectively) each displayed intense diffraction peaks in the lower 2*θ* region, indicative of relatively high crystallinity of the as-prepared frameworks. However, the XRD patterns hinted at potential differences in the stacking of the two-dimensional sheets between the three samples. The patterns from A-CuHHTP and C-CuHHTP showed relatively good agreement with simulated XRD patterns of Cu_3_(HHTP)_2_ subunits with both the *P*6/*mmm* space group, with eclipsed cofacial AA-stacking of the layers, and the *C*2/*m* space group, with near-eclipsed staggered AA-stacking (*i.e.*, a constant stacking shift of the sheets) (Fig. 2c, d and SI Table S1†). Broadening of the diffraction peaks, potentially due to the presence of stacking defects, prevented distinguishing between the two models for these samples. However, the diffraction pattern of B-CuHHTP showed significantly better agreement with that of the near-eclipsed stacking model. This can be clearly seen by examining the low 2*θ* diffraction peaks. Distinct peak splitting is seen in this region for B-CuHHTP, closely resembling the peak splitting observed in the simulated pattern for the near-eclipsed model (Fig. 2b). This splitting was not conclusively seen in the diffraction patterns of either A-CuHHTP or C-CuHHTP. This suggests a potential increased stacking shift of the layers when Cu_3_(HHTP)_2_ is synthesised in the presence of DMF, consistent with previous observations.^17^ This indicates that modulator identity can influence the stacking sequence of these materials, an exciting phenomenon which has not been noted previously. Future work is needed to further examine the impact of modulator identity on the structures of layered MOFs.

**Fig. 3:**
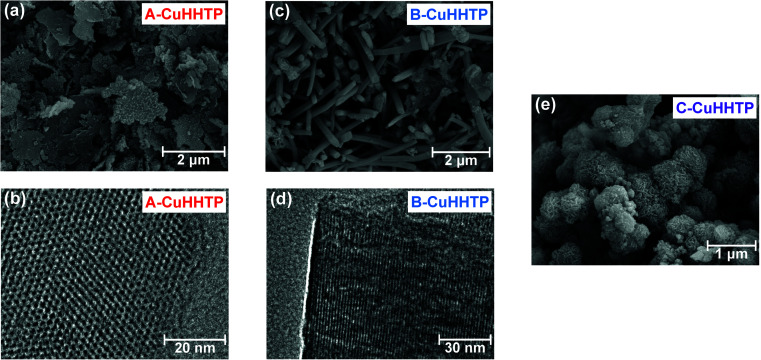
(a) SEM and (b) TEM images of A-CuHHTP; (c) SEM and (d) TEM images of B-CuHHTP; and (e) SEM images of C-CuHHTP. These illustrate the differences in microstructure and three-dimensional pore network between the samples.

Following crystal structure characterisation, scanning electron microscopy (SEM) images confirmed differences in microstructure. A-CuHHTP was formed of weakly agglomerated ‘flake-like’ particles, with diameters of 0.5–2.9 μm and thicknesses of 30–110 nm based on measurements on a range of different particles seen in SEM images (Fig. 3a and SI Fig. S1, Table S2†).^18^ This gives a length-to-width aspect ratio for this sample of 0.01–0.23. Transmission electron microscopy (TEM) confirmed that the pores run perpendicular to the face of the flakes, showing that particles of A-CuHHTP have a pore network made up of many short pores (Fig. 3b and SI Fig. S2†). This illustrates that using ammonia as a modulator in the synthesis of Cu_3_(HHTP)_2_ leads to inhibited crystal growth in the *c* crystallographic direction and enhanced growth in the *ab* plane. In marked contrast, samples of B-CuHHTP showed weakly agglomerated ‘rod-like’ particles, with diameters of 110–340 nm and lengths of 0.5–4 μm observed in SEM images (Fig. 3c and SI Fig. S3, Table S3†). TEM images, and the determination of the fringe spacing in these, confirmed that the pores run parallel to the length of the rods (Fig. 3d). This verifies that employing DMF as a modulator leads to a significant increase in growth in the *c* crystallographic direction, with a length-to-width aspect ratio of 1–37 estimated from SEM images.^17^ This results in a pore network of relatively long pores and few pore openings, contrasting with that of A-CuHHTP. Interestingly, comparing the fast Fourier transform (FFT) image from this sample with simulated FFT images from Cu_3_(HHTP)_2_ with both eclipsed and near-eclipsed stacking sequences supports the XRD analysis that B-CuHHTP has near-eclipsed layer stacking (SI Fig. S4†). While both A-CuHHTP and B-CuHHTP displayed weak particle agglomeration, C-CuHHTP exhibited a strongly agglomerated microstructure of *ca.* 0.5–1 μm spherical clusters of nanoscale ‘flake-like’ particles (Fig. 3e). Due to the thickness of the agglomerates, TEM imaging was unable to examine the pore network in this sample. These results confirm that coordination modulation is an effective means to reliably obtain layered MOFs with distinctly different sample microstructures. It must be noted, however, that all samples displayed relatively large polydispersities, with a range of length-to-aspect ratios observed for the crystallites in each sample. Future work should focus on developing new synthesis methods to produce samples with more uniform particle dimensions.

To further assess differences in the physical properties of the samples, additional characterisation was conducted. Electrical conductivity measurements showed no obvious differences with a value of approximately 0.002 S cm^−1^ recorded for each sample, within the literature range for polycrystalline samples of Cu_3_(HHTP)_2_ (SI Table S4†).^3,15,29^ Elemental analysis showed that each sample had approximately the expected stoichiometric ratio of Cu to HHTP, further demonstrating successful synthesis of the framework with each modulator (SI Table S5†). However, nitrogen-containing impurities were present in both A-CuHHTP and C-CuHHTP. Importantly, extended washing of these samples (*ca.* 14 days in MeOH under N_2_) resulted in negligible reduction in nitrogen content. This suggests that the nitrogen-containing impurities, hypothesised to be small amounts of ammonia and pyridine, respectively, are incorporated into the framework during crystal formation. As noted above, additional work is required to understand the growth mechanisms of layered conductive MOFs in more detail, including why certain modulators are incorporated into the crystal structures of layered MOFs.

Finally, the porosity and Brunauer–Emmett–Teller (BET) areas were evaluated using 77 K N_2_ sorption isotherms (SI Fig. S5†). BET areas of 759 ± 32, 314 ± 11, and 293 ± 33 m^2^ g^−1^ were recorded for A-CuHHTP, B-CuHHTP, and C-CuHHTP, respectively. The BET area for A-CuHHTP is in line with that reported previously for this material, while these are the first reported values for B-CuHHTP and C-CuHHTP.^11^ These results show that A-CuHHTP has a greater nitrogen-accessible porosity than both B-CuHHTP and C-CuHHTP. Pore size distribution analysis showed that all samples had well-defined pore sizes of 14–16 Å (SI Fig. S6†). This agrees well with the crystallographically expected value of 16 Å, and illustrates that all samples have the anticipated micropore structure.

Following characterisation of the samples, electrode films composed of Cu_3_(HHTP)_2_, acetylene black and PTFE in the ratio 85 : 10 : 5 were fabricated, as in previous studies.^22,30^ SEM imaging confirmed that the microstructures observed in powdered samples were broadly maintained following film formation (SI Fig. S7†). The energy storage performances of A-CuHHTP, B-CuHHTP, and C-CuHHTP were then investigated by assembling symmetric supercapacitor cells with both an organic electrolyte, 1 M NEt_4_BF_4_ in acetonitrile (ACN), and an ionic liquid electrolyte, EMIM-BF_4_. All cells with a given electrolyte were assembled with similar electrode mass loadings where possible to allow for fair comparison, and the contributions of both acetylene black and PTFE to the capacitive performance were removed (SI Table S6 and Fig. S8†). Neat pellets of Cu_3_(HHTP)_2_ displayed poor performances in supercapacitors, most likely caused by the lower electrical conductivity of Cu_3_(HHTP)_2_ compared to similar layered frameworks, and thus could not be used in this study (SI Fig. S9†).

**Fig. 4:**
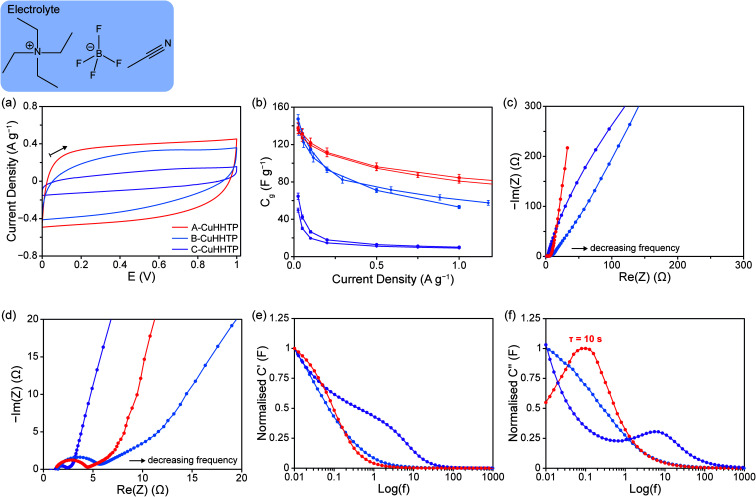
Characterisation data from symmetric supercapacitors assembled with A-CuHHTP, B-CuHHTP, and C-CuHHTP composite electrodes and NEt_4_BF_4_/ACN electrolyte. (a) CVs obtained at a scan rate of 10 mV s^−1^ up to a final cell voltage of 1 V, with the arrow indicating the direction of scanning. (b) Specific capacitance *versus* current density graphs from charging up to 1 V, with results shown for two independent cells for each of the three materials. *C*_*g*_ values were determined using only the mass of active Cu_3_(HHTP)_2_ material in the supercapacitors. (c) Nyquist plots from EIS, and (d) zoomed view to show the high and intermediate frequency domains in greater detail. Plots of (e) normalised *C*′ against log(*f*), and (f) normalised *C*′′ against log(*f*), calculated from EIS data.

With 1 M NEt_4_BF_4_/ACN electrolyte, cyclic voltammetry (CV) experiments at a scan rate of 10 mV s^−1^ showed quasi-rectangular traces and confirmed a stable voltage window of *ca.* 1 V for all samples of Cu_3_(HHTP)_2_, consistent with our previous work for this layered framework (Fig. 4a).^11^ However, voltammograms from B-CuHHTP and C-CuHHTP exhibited consistently smaller areas, indicating lower charge storage on this timescale for samples with either ‘rod-like’ or strongly agglomerated particles relative to those with weakly agglomerated ‘flake-like’ particles. Galvanostatic charge–discharge (GCD) experiments between 0–1 V at a range of current densities further probed the performances of the three samples.

Despite the initial CV results, A-CuHHTP and B-CuHHTP exhibited similar specific capacitances, *C*_*g*_, at low current densities (129 ± 2 and 127 ± 5 F g^−1^, respectively) (Fig. 4b). This suggests that both A-CuHHTP and B-CuHHTP have pore structures which are freely accessible to organic electrolyte ions at long charging times. This result seemingly contradicts the N_2_ sorption isotherm data, which suggested a larger accessible surface area for A-CuHHTP. As reported previously, this suggests that BET areas are not always an accurate predictor of the electrolyte ion accessible surface area, and therefore the capacitance, of porous materials.^31,32^ A possible reason for the lower BET area of B-CuHHTP is that residual solvent molecules or other impurities may be partially blocking the pores of the ‘rod-like’ crystals. Such blockages would have a more significant impact on the measured BET area of B-CuHHTP than A-CuHHTP due to the different average pore lengths of the two samples. However, diffusion of ions during electrochemical cycling may displace these solvent molecules, freeing up surface area that was previously inaccessible. This would result in similar electrochemically accessible surface areas for the two samples, and thus similar energy storage performances at slow charging rates. In contrast, C-CuHHTP displayed a significantly lower specific capacitance of 36 ± 6 F g^−1^ at 0.05 A g^−1^, supporting previous reports that strongly agglomerated microstructures have lower energy storage performances compared to samples with weaker particle agglomeration.^22,23^ This is likely due to limited ionic diffusion through the spherical agglomerates, resulting in poor ion accessibility to the crystallites at the centre of the agglomerates.

Cells constructed with B-CuHHTP consistently recorded lower rate capabilities across the same current density range (*C*_*g*_ retention of 40 ± 4% between 0.025–1 A g^−1^) than those assembled with A-CuHHTP (*C*_*g*_ retention of 60 ± 1%) (Fig. 4b and SI Fig. S10†). This is consistent with the results from CV experiments above, which have current densities of between 0.3–0.5 A g^−1^, and demonstrates that short charging times lead to poor energy storage in ‘rod-like’ particles. Consideration of the different pore structures of the two samples can explain this difference in behaviour. For the ‘flake-like’ particles of A-CuHHTP, the pores run perpendicular to the face of the flakes, resulting in many short pores. This creates an easily accessible pore network with short diffusion paths through the particles. In contrast, the ‘rod-like’ particles of B-CuHHTP have pores which run parallel along the length of the crystals, resulting in fewer, longer pores. This reduces accessibility to the full pore network at short charging times as the diffusion paths through the particles are longer. As a result, lower current densities and longer charging times are needed to allow electrolyte ions to access the entire pore volume in B-CuHHTP. C-CuHHTP also displays a very low rate capability over the same current density range (17 ± 2%), supporting the above conclusion that strong agglomeration of the MOF particles further reduces pore accessibility.

To test the above hypothesis, electrochemical impedance spectroscopy (EIS) was performed on the cells (Fig. 4c, d and SI Fig. S11†). Typically, the Nyquist plots of supercapacitors show three distinct domains. (i) At high frequencies, the supercapacitor behaves as a pure resistor and a semi-circular response may be seen depending on cell assembly. The radius of the semi-circle and its initial intercept with the real axis primarily depend on the contact and bulk electrolyteresistances, although some authors state that charge-transfer resistance can also contribute to this domain.^33–35^ (ii) The intermediate frequency domain is characterised by a linear line at *ca.* 45° relative to the Re(*Z*) axis, which has been assigned to the penetration of the electrolyte into the pore network of the electrode and the corresponding transition to diffusion-limited electrolyte transport. A shift of this domain to lower frequencies and higher real impedances suggests more limited ionic mass transport and slower electric double-layer formation.^36,37^ (iii) At low frequencies, a linear response is observed, attributed to the capacitive behaviour of the electric double-layer formed at the electrode/electrolyte interface. An ideal supercapacitor displays a vertical line in this domain, and the extent of variation from this theoretical response indicates non-ideal capacitive behaviour, which may bedue to poor ionic accessibility and movement.^33,36,38^ Curvature of the response in this domain can indicate a high resistance to ion movement within the electrode pores or faradaic contributions to charge storage.

Cells constructed with both A-CuHHTP and B-CuHHTP exhibited similar high frequency responses as expected, with semi-circular responses and measured internal resistances of *ca.* 7.3 and 6.2 Ω, respectively. However, those constructed with B-CuHHTP displayed a low-frequency response with a greater variation from the idealised vertical line. This indicates poorer movement of the electrolyte ions through the ‘rod-like’ crystallites of B-CuHHTP than the ‘flake-like’ crystallites of A-CuHHTP. This supports the above hypothesis and confirms that ‘flake-like’ particle morphologies, as in A-CuHHTP, are desirable for higher capacitive performance with organic electrolytes, with the pore network allowing for easier access and motion of electrolyte ions. This supports previous observations with two-dimensional porous carbon nanosheets.^39,40^

Additional support for this hypothesis is seen by examining how both the real capacitance (*C*′) and imaginary capacitance (*C*′′) vary with frequency (Fig. 4e and f). For a supercapacitor, the plot of *C*′ against log(*f*) tends to a plateau at low frequencies as the maximal capacitance is reached.^35,36^ Failure to plateau suggests incomplete ion accessibility of the pore volume and imperfect charge storage. The plot of *C*′′ against log(*f*) typically goes through a maximum at a characteristic frequency, *f*_*o*_. This defines a time constant *τ*_*o*_, the characteristic relaxation time of the system (*i.e.*, the minimum time to discharge all of the energy from a device with an efficiency of more than 50%).^35,41^ A shift of *τ*_*o*_ to higher values suggests slower ionic movement in the material. Plots of *C*′ and *C*′′ against log(*f*) for A-CuHHTP and B-CuHHTP confirmed lower ionic accessibility and movement through the pore structure of the ‘rod-like’ crystals, with B-CuHHTP failing to reach a *C*′ plateau or exhibit a maximum in *C*′′. This supports the above Nyquist plot interpretation. Future work to directly observe the diffusion of NEt_4_BF_4_ within each sample, as has been performed previously for porous carbons, would further confirm these findings.^41–43^ Finally, while cells assembled with C-CuHHTP displayed low internal resistances on-par with those of A-CuHHTP and B-CuHHTP (2.5 Ω), significant curvature of the high frequency response was observed. This is further evidence for high resistance to ion movement through agglomerated microstructures.

**Fig. 5:**
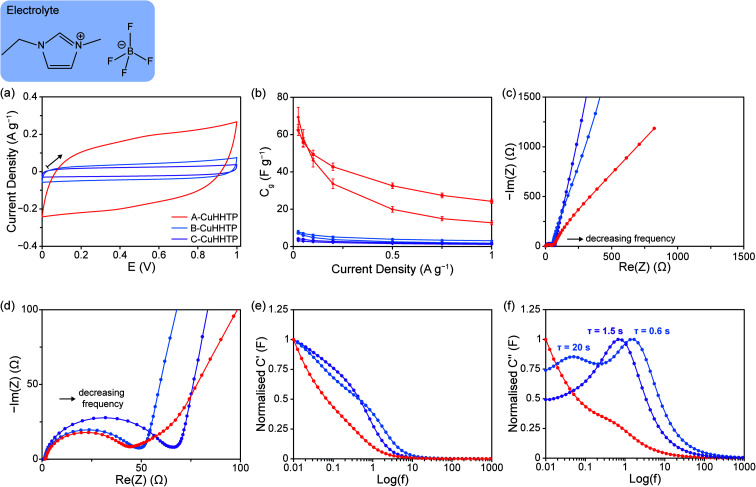
Characterisation data from symmetric supercapacitors assembled with A-CuHHTP, B-CuHHTP, and C-CuHHTP composite electrodes and EMIM-BF_4_ electrolyte. (a) CVs obtained at a scan rate of 10 mV s^−1^ up to a final cell voltage of 1 V, with the arrow indicating the direction of scanning. (b) Specific capacitance *versus* current density graphs from charging up to 1 V, with results shown for two independent cells for each of the three materials. *C*_*g*_ values were determined using only the mass of active Cu_3_(HHTP)_2_ material in the supercapacitors. (c) Nyquist plots from EIS, and (d) zoomed view to show the high and intermediate frequency domains in greater detail. Plots of (e) normalised *C*′ against log(*f*), and (f) normalised *C*′′ against log(*f*), calculated from EIS data.

To examine the influence of sample microstructure with a wider range of commercially-relevant electrolytes, cells were assembled with undiluted EMIM-BF_4_ (6.3 M), an ionic liquid commonly used in supercapacitors. Similar to experiments with organic electrolytes, CV experiments with EMIM-BF_4_ at 10 mV s^−1^ confirmed a stable voltage window of *ca.* 1 V for all Cu_3_(HHTP)_2_ samples (Fig. 5a), and B-CuHHTP and C-CuHHTP once again exhibited lower charge storage on this timescale. However, GCD experiments revealed notable differences in behaviour with EMIM-BF_4_ compared to the organic electrolyte. While a specific capacitance of 57 ± 2 F g^−1^ was recorded for A-CuHHTP at low current densities, similar to previously reported values for related frameworks with this electrolyte, both B-CuHHTP and C-CuHHTP displayed significantly lower specific capacitances of 8 ± 2 and 3 ± 1 F g^−1^, respectively (Fig. 5b).^44^ This is in contrast to the performance with organic electrolytes, where both A-CuHHTP and B-CuHHTP exhibited similar specific capacitances at low current densities. To determine if this was a kinetic effect, cells were charged at a very low current density of 0.0025 A g^−1^ to allow additional time for ionic diffusion and exchange (SI Fig. S12 and Table S6†). Once more, the specific capacitance of A-CuHHTP was much greater than both B-CuHHTP and C-CuHHTP. Strikingly, this suggests that there is significantly lower penetration of EMIM^+^ and BF_4_^−^ ions into the pore structures of these samples compared to A-CuHHTP. This confirms the superior performance of ‘flake-like’ particle morphologies with pure ionic liquid electrolytes.

EIS studies were used to probe the accessibility of EMIM-BF_4_ into the MOF electrodes by examining the intermediate frequency domain. Whilst this domain is clearly identifiable in the EIS spectrum of A-CuHHTP, with a transition frequency of 3.4 Hz, it is almost absent from the spectra of B-CuHHTP and C-CuHHTP (Fig. 5c and d). This unusual behaviour, which was reproducible for three independent cells (SI Fig. S13†), combined with the low observed capacitances, suggests that movement of the ionic liquid through the pore networks of these two samples is severely impeded. This leads to a high proportion of EMIM^+^ and BF_4_^−^ ion electrosorption occurring on the external surface area for B-CuHHTP and C-CuHHTP, limiting charge storage in these systems and leading to low capacitances (Fig. 5b). Further support for this hypothesis is seen in plots of *C*′′ against log(*f*) (Fig. 5e and f). Short *τ*_o_ values of 0.6 and 1.5 s are recorded for B-CuHHTP and C-CuHHTP, respectively, consistent with rapid discharge from particle surfaces. The differences in microstructure discussed above, together with the increased viscosity of the ionic liquid, are the likely cause of the lower ionic accessibility and mobility of EMIM-BF_4_ for these samples. Future work to examine the adsorption of ions within these porous materials is needed to confirm this hypothesis.

## 3 Conclusions

In this study, we successfully showed that sample microstructure has a significant impact on the energy storage performance of the layered MOF Cu_3_(HHTP)_2_. Weakly agglomerated ‘flake-like’ particles displayed superior energy storage performances in supercapacitors with both organic and ionic liquid electrolytes compared with samples with either weakly agglomerated ‘rod-like’ or strongly agglomerated particles. GCD and EIS studies confirmed that this is due to improved ion accessibility and dynamics within the pore structure of the ‘flake-like’ particles, which have many short pores. The use of coordination modulation enabled a much greater variation in microstructure than was possible in previous work on this topic, with a variation in the length-to-aspect ratio from 0.01 to 37 recorded in this work. Therefore, our work is the first to comprehensively reveal the impact of sample microstructure on the energy storage performances of layered MOFs.

Ultimately, these results show that weakly agglomerated ‘flake-like’ particle morphologies with very small length-to-width aspect ratios (<1) are best for MOF supercapacitors. This result will guide the synthesis of future MOFs for a wide range of energy storage applications.

## Data availability

All raw experimental data files are available in the Cambridge Research Repository, Apollo, with the identifier https://doi.org/10.17863/CAM.82440.

## Author contributions

J. W. G. and A. C. F. designed the research. J. W. G. performed and analysed the SEM, gas sorption, electrical conductivity and electrochemistry experiments. J. W. G and C. J. B. performed and analysed the XRD experiments. J. W. G. and S. M. F. performed and analysed the TEM experiments. All authors interpreted the results and contributed to the writing of the manuscript.

## Conflicts of interest

There are no conflicts to declare.

## Supplementary Material

SC-013-D2SC03389E-s001
