# Supplementary material for: Enhancing the energy storage performances of metal–organic frameworks by controlling microstructure
Source: Chem Sci. 2022 Jul 18;13(32):9210–9. doi: 10.1039/d2sc03389e (PMC9384154; doi:10.1039/d2sc03389e)
Supplement: SC-013-D2SC03389E-s001 [file SC-013-D2SC03389E-s001.pdf]

Electronic Supplementary Material (ESI) for Chemical Science.
This journal is © The Royal Society of Chemistry 2022

Supplementary Information for:

Enhancing the Energy Storage Performances of Metal-Organic Frameworks by Controlling Microstructure

Jamie W. Gittins,[1] Chloe J. Balhatchet,[1] Simon M. Fairclough,[2] and Alexander C. Forse[1*]

[1] Yusuf Hamied Department of Chemistry, University of Cambridge, Lensfield Road, Cambridge CB2 1EW, U.K.

[2] Department of Materials Science & Metallurgy, University of Cambridge, 27 Charles Babbage Road, Cambridge CB3 0FS, U.K.

*acf50@cam.ac.uk

Contents

Materials ... 2
Synthesis ... 2
  A-CuHHTP, C-CuHHTP ... 2
  B-CuHHTP ... 3
Materials Characterisation ... 3
  X-ray Diffraction (XRD) ... 3
  Scanning Electron Microscopy (SEM) ... 3
  Transmission Electron Microscopy (TEM) ... 3
  Electrical Conductivity Measurements ... 4
  Elemental Analysis ... 4
  Gas Sorption ... 4
Electrochemical Characterisation ... 4
  Electrode Preparation ... 4
  Supercapacitor Assembly ... 5
  Electrochemical Cell Characterisation ... 5
Table S1 ... 7
Figure S1 ... 8
Table S2 ... 9
Figure S2 ... 10
Figure S3 ... 11
Table S3 ... 12
Figure S4 ... 13
Table S4 ... 14
Table S5 ... 15
Figure S5 ... 16
Figure S6 ... 17
Figure S7 ... 18
Table S6 ... 19
Figure S8 ... 20
Figure S9 ... 21
Figure S10 ... 22
Figure S11 ... 23
Figure S12 ... 24
Table S7 ... 25
Figure S13 ... 26
Supplementary References ... 27

Materials

All materials were purchased from commercial suppliers, used without additional modification and handled in air unless specified below.

Tetraethylammonium tetrafluoroborate (NEt_4BF_4) was dried under vacuum at 100 °C for 48 h before being transferred to a N_2-filled glovebox. Anhydrous acetonitrile (ACN) was purged with N_2 for 3 h before taking it into a N_2-filled glovebox, where it was further dried by the addition of activated 3 Å molecular sieves. Molecular sieves were activated at 250 °C in a vacuum oven for 12 h prior to transferring into a N_2-filled glovebox. 1-Ethyl-3-methylimidazolium tetrafluoroborate (EMIM-BF_4) was dried at room temperature under dynamic vacuum for 120 h before being transferred to a N_2-filled glovebox.

Any unexpected observations and safety hazards are noted below.

Synthesis

$Cu_3(HHTP)_2$ samples were synthesised with three different modulators: ammonia, N,N-dimethylformamide (DMF), and pyridine.

A-CuHHTP, C-CuHHTP

Ammonia and pyridine-modulated $Cu_3(HHTP)_2$ samples (A-CuHHTP and C-CuHHTP, respectively) were synthesised by modifying a recently published literature procedure.[1] For A-CuHHTP, 35% aqueous ammonia solution was used as the source of the modulator. For C-CuHHTP, pyridine was used as the modulator.

A solution of $Cu(NO_3)_2.3H_2O$ (0.127 g, 0.526 mmol, 1.65 eq) and the modulator (50 eq) in distilled water (2 mL) was prepared. The resulting royal blue solution was added dropwise to a dispersion of H_6HHTP (0.103 g, 0.318 mmol, 1.00 eq) in distilled water (8.2 mL) in a 40 mL screw-top vial. The vial was closed with a screw cap fitted with a septum and the resulting mixture was heated in a furnace oven at 80 °C for 24 h. The dark blue precipitate formed was separated by centrifugation and the supernatant layer discarded. The precipitate was then washed successively with water (3 × 30 mL), ethanol (3 × 30 mL), and acetone (3 × 30 mL). Washing was performed by centrifuging the precipitate with the desired washing solvent for 15 minutes before removing the supernatant layer, replacing with fresh washing solvent, shaking the centrifuge tube vigorously, and centrifuging once again. No soaking of the precipitate was performed. The precipitate was then filtered by vacuum filtration, and the resulting dark blue powder was dried at 80 °C under dynamic vacuum for 96 h and then stored in a N_2-filled glovebox until used. No differences in the physical appearances of the reaction mixtures and products were observed between the syntheses of A-CuHHTP and C-CuHHTP.

B-CuHHTP

DMF-modulated $Cu_3(HHTP)_2$ (B-CuHHTP) was synthesised by modifying a recently published literature procedure.[2]

H_6HHTP (0.117 g, 0.361 mmol, 1.00 eq) was added to a mixture of 1.5 mL (19.5 mmol, 54.0 eq) of DMF in 12 mL of H_2O. The resulting solution was sonicated for 15 mins at room temperature, leading to partial dissolution of the solid and the formation of a dark brown solution. A solution of $CuSO_4.5H_2O$ (0.205 g, 0.820 mmol, 2.27 eq) in distilled water (9 mL) was also prepared. Both solutions were heated in an oven at 80 °C for 10 mins prior to being mixed. The 40 mL vial was closed with a screw cap fitted with a septum, and the resulting mixture was then heated at 80 °C for 12 h. The dark blue precipitate formed was separated by centrifugation and the supernatant layer discarded. The precipitate was then washed successively with water (3 × 30 mL), ethanol (3 × 30 mL), and acetone (3 × 30 mL). Washing was performed as stated above. The precipitate was filtered by vacuum filtration and washed further on the filter with water (250 mL), ethanol (250 mL) and acetone (250 mL). The resulting dark blue powder was dried at 80 °C under dynamic vacuum for 96 h and then stored in a N_2-filled glovebox until used.

Materials Characterisation

X-ray Diffraction (XRD)

High resolution synchrotron XRD data were collected at the I11 beamline at Diamond Light Source. Samples were loaded into borosilicate glass capillary tubes (0.5 mm outside diameter, 0.01 mm wall thickness; Capillary Tube Supplies Ltd.) in a N_2-filled glovebox, and then sealed with Loctite EA 3430 epoxy adhesive. The adhesive was allowed to cure in the glovebox at ambient temperature for at least 72 h before removal of the capillaries. Diffraction patterns were collected under ambient conditions using a Mythen II position-sensitive detector (PSD) with two 5-second scans separated by an angular shift in detector position of 2.5 °. The wavelength and intrinsic peak-shape parameters were refined against a known Si 640c NIST standard. The refined wavelength for the PSD scans was 0.82683 Å (~ 15 keV). Simulated XRD patterns were produced using VESTA version 3.[3] Computational structures used to produce the simulated PXRD patterns are available at: https://doi.org/10.5281/zenodo.4694845. Crystal structures were visualised in VESTA version 3.

Scanning Electron Microscopy (SEM)

SEM imaging was performed on a Tescan MIRA3 FEG-SEM. Samples were secured onto stainless-steel SEM stubs using adhesive high purity carbon tabs, and then sputter-coated with a thin layer (~ 5 nm) of Pt using a Quorum Technologies Q150T ES Turbo-Pumped Sputter Coater. Imaging was conducted with a beam voltage of 5 kV and working distances of 4 – 8 mm. Analysis of SEM images was performed in FIJI ImageJ (https://imagej.net/Fiji/).[4]

Transmission Electron Microscopy (TEM)

TEM imaging was performed using Thermo Fisher Scientific F20 operating with at 200 keV with a beam flux of < 30 e^- / $Å^2$ s. Images were taken using a Gatan OneView 4k Camera with an acquisition time of 0.5 s. The sample was aligned off the region of interest and images were taken immediately when on the region of interest to avoid the impacts of electron beam damage. Analysis of TEM images was performed in FIJI ImageJ. FFT image simulations were performed using CrystalMaker software.

Electrical Conductivity Measurements

The electrical conductivity of $Cu_3(HHTP)_2$ samples was measured via a two-point probe method using a homemade set-up. Pellets composed of $Cu_3(HHTP)_2$ were prepared by loading the material into a 13 mm Evacuable Pellet Die (Specac) and applying a force of 4 ton-force cm^{-2} for 1 min with a Specac hydraulic press. The areal mass loading of the pellets was approximately 50 mg cm^{-2}, and the thickness varied between 200 – 300 µm. Samples were pressed between two stainless-steel electrodes using a hydraulic press (Specac), with PTFE disks used to prevent short-circuiting through the press. All measurements were conducted with a loading of 4 ton-force cm^{-2}. Resistances were measured using a Keithley 2000 Multimeter. The electrical conductivity, σ (S cm^{-1}), of the sample was calculated according to: $\sigma = L/RA$, where L is the thickness of the sample (cm), A is the area of the sample (cm^2), and R is the measured resistance (Ω). All values of L and A were measured following completion of the measurement, assuming a non-elastic material. The primary source of error in the measurement was determining the thickness of the pellets.

Elemental Analysis

Cu content was determined via inductively coupled plasma optical emission spectroscopy using a Thermo Scientific iCAP-7400 ICP spectrometer. C, H and N concentrations were determined via CHN combustion analysis using an Exeter Analytical CE-440, with combustion at 975 °C.

Gas Sorption

Low pressure N_2 isotherms (adsorption and desorption) were collected using an Anton Parr Autosorb iQ-XR at 77 K. An oven-dried sample cell (Type A long cell, 9 mm outer diameter, LG bulb) was tared before being loaded with the sample. Ex-situ degassing (80 °C, 24 h) was performed before the evacuated tube was weighed again to determine the sample mass. Isotherms were collected over 24 – 30 h, and the samples reweighed following analysis to ensure accurate mass readings. Sorption isotherms were evaluated in AsiQwin version 5.21 software. All $Cu_3(HHTP)_2$ samples displayed Type I N_2 isotherms, with high gas uptake below 0.1 P/Po, indicating microporosity. Material BET areas were calculated from isotherms using the BET equation and Rouquerol's consistency criteria implemented in AsiQwin.[5–7] All pore size distribution fittings were conducted in AsiQwin using N_2 at 77 K on carbon (cylindrical pores) quenched solid density functional theory (QSDFT) model with a bin pore width of 0.5 Å.

Electrochemical Characterisation

Electrode Preparation

Freestanding composite $Cu_3(HHTP)_2$ films were prepared using an existing literature method.[1] The masses of components were calculated so that the final films had a composition of 85 wt% $Cu_3(HHTP)_2$, 10 wt% acetylene black (nominal surface area stated by supplier = 75 m^2 g^{-1}, measured BET area = 62 m^2 g^{-1}), and 5 wt% PTFE. The same procedure was used for all $Cu_3(HHTP)_2$ samples, and all films had

a thickness of *ca.* 250 μm. Films made with acetylene black as the only active material were prepared using the same method.

Neat $Cu_3(HHTP)_2$ pellet electrodes were prepared using the same method as described above (see 'Electrical Conductivity Measurements' section).

Supercapacitor Assembly

Symmetric supercapacitors were prepared as coin cells in Cambridge Energy Solutions CR2032 SS316 coin cell cases. Film electrodes were cut from freestanding composite $Cu_3(HHTP)_2$ and acetylene black films with areal mass loadings ranging between 7 – 18 mg cm^{-2}. Pellet electrodes were used as prepared. The electrodes were dried in vacuo at 80 °C for at least 24 h prior to assembling the cell in a N_2-filled glovebox. A 1 M solution of NEt_4BF_4 in ACN and undiluted EMIM-BF_4 ionic liquid (6.6 M) were used as electrolytes. The volume of electrolyte added was consistent between cells (10 drops from a Pasteur pipette). Whatman glass microfiber filter (GF/A) was used as a separator. This was dried in vacuo at 100 °C for 24 h prior to use. Each coin cell contained two SS316 separator disks and one SS316 spring to ensure sufficient and consistent pressure in the cells. The coin cells were sealed in the glovebox using a Compact Hydraulic Coin Cell Crimper (Cambridge Energy Solutions) before being removed for testing.

Electrochemical Cell Characterisation

All electrochemical measurements were carried out using a Biologic SP-150 potentiostat and a Biologic BCS-800 Series ultra-precision battery cycler. EIS measurements were performed in the frequency range from 1 MHz to 10 mHz (decreasing frequency) at the open circuit voltage (OCV) using a single-sinusoidal signal with a sinus amplitude of 10 mV and drift correction applied. The specific capacitance, C_g (F g^{-1}), was calculated from GCD discharge profiles using the *Supycap* Python code (GitHub - AdaYuanChen/Supycap: Analysis tool for the CC and CV experiment of supercapacitors). C_g values were determined using only the mass of active $Cu_3(HHTP)_2$ material in the supercapacitors.

The internal resistance, *R,* was calculated from both Nyquist plots produced from EIS measurements, and from the voltage drop at the beginning of GCD discharge profiles. For the calculation from Nyquist plots, *R* was obtained from the low-frequency interception of the semi-circular response with the *Re(Z)* axis, as in the literature.[8] For the calculation from GCD discharge profiles, the *Supycap* Python code was used.

Current densities were calculated by dividing the current applied during the GCD experiment, *I*, by the average mass of active MOF material per electrode, $\bar{m}$.

C' and *C"* were calculated from EIS data using the following equations:

$$C'(\omega) = {-Z''(\omega)}/{\omega|Z(\omega)|^2} \quad [1]$$

$$C''(\omega) = {Z'(\omega)}/{\omega|Z(\omega)|^2} \quad [2]$$

Where C' is the real capacitance (F), C'' is the imaginary capacitance (F), ω is the angular frequency (rad), $Z'(\omega)$, also written as $Re(Z)$, is the real impedance (Ω), $Z''(\omega)$, also written as $Im(Z)$, is the imaginary impedance (Ω), and $Z(\omega)$ is the total impedance (Ω).

Table S1: Crystallographic Data for Simulated $Cu_3(HHTP)_2$ Structures

	Structure 1	Structure 2
Crystal System	Hexagonal (Eclipsed)	Monoclinic (Near-eclipsed)
Space Group	*P*6/*mmm*	*C*2/*m*
a / Å	21.50	37.98
b / Å	21.50	21.93
c / Å	3.20	3.31
α / °	90	90
β / °	90	74.80
γ / °	90	90
Volume of Unit Cell / $Å^3$	1281	2660

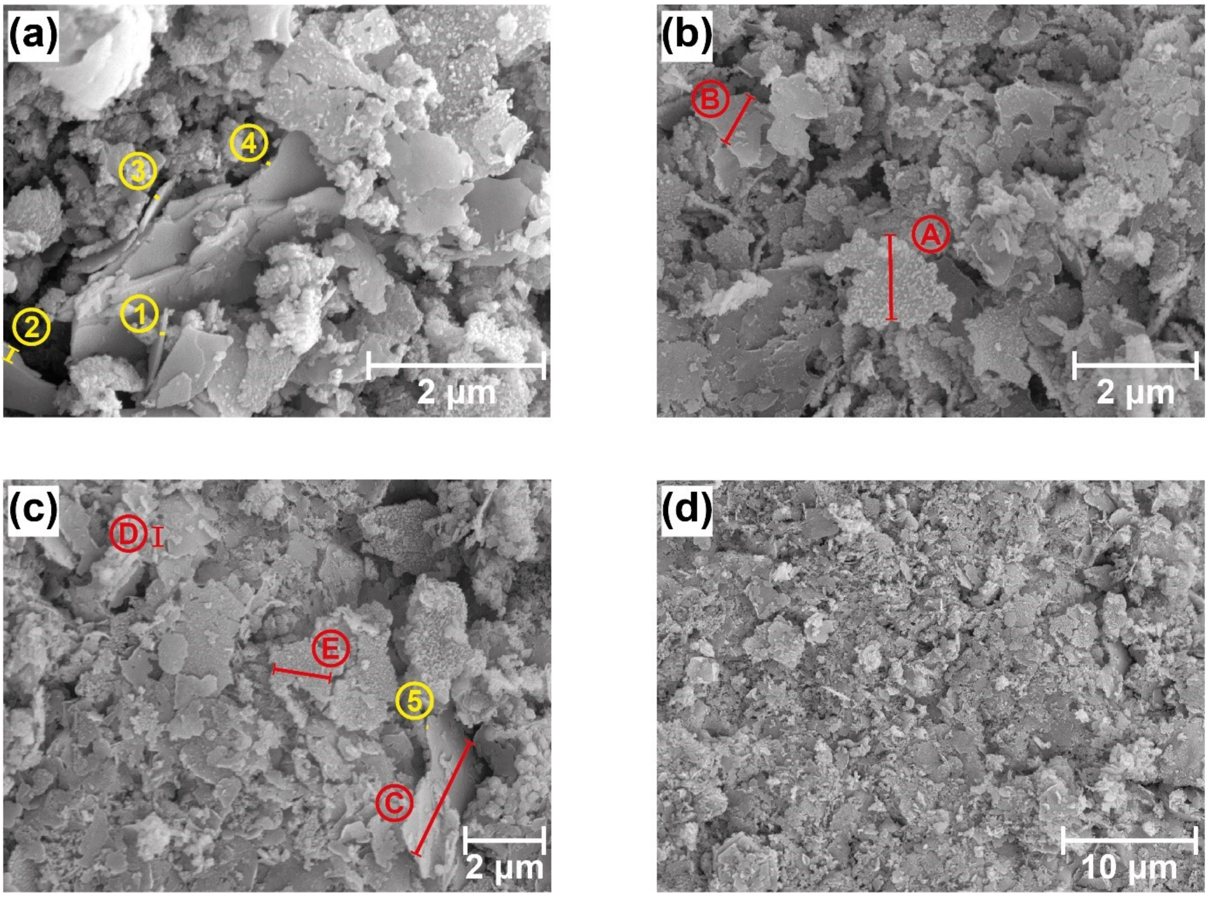

Figure S1: Selection of SEM images from a range of A-CuHHTP samples. The measurements shown (summarised in Table S2 below) were used to determine the length-to-aspect ratio of the 'flake-like' particles.

Table S2: Determination of Length-to-Width Aspect for A-CuHHTP from SEM Images

Label	Dimension	Measurement / μm
1	Length	0.05
2	Length	0.11
3	Length	0.04
4	Length	0.06
5	Length	0.03
A	Width	1.27
B	Width	0.75
C	Width	2.86
D	Width	0.48
E	Width	1.67

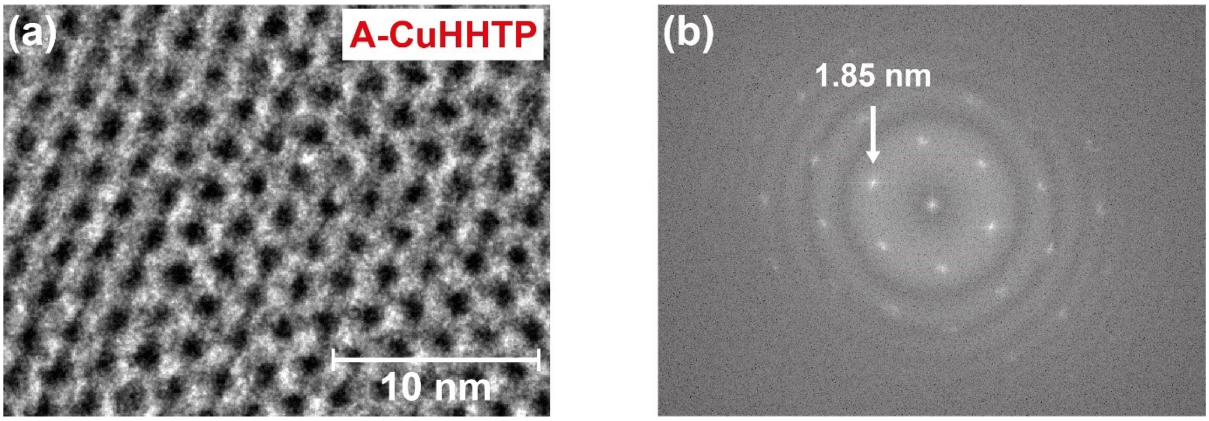

Figure S2: (a) TEM image of a ‘flake-like’ particle of A-CuHHTP, imaged perpendicular to the face of the particles. (b) Corresponding fast Fourier transform (FFT) image.

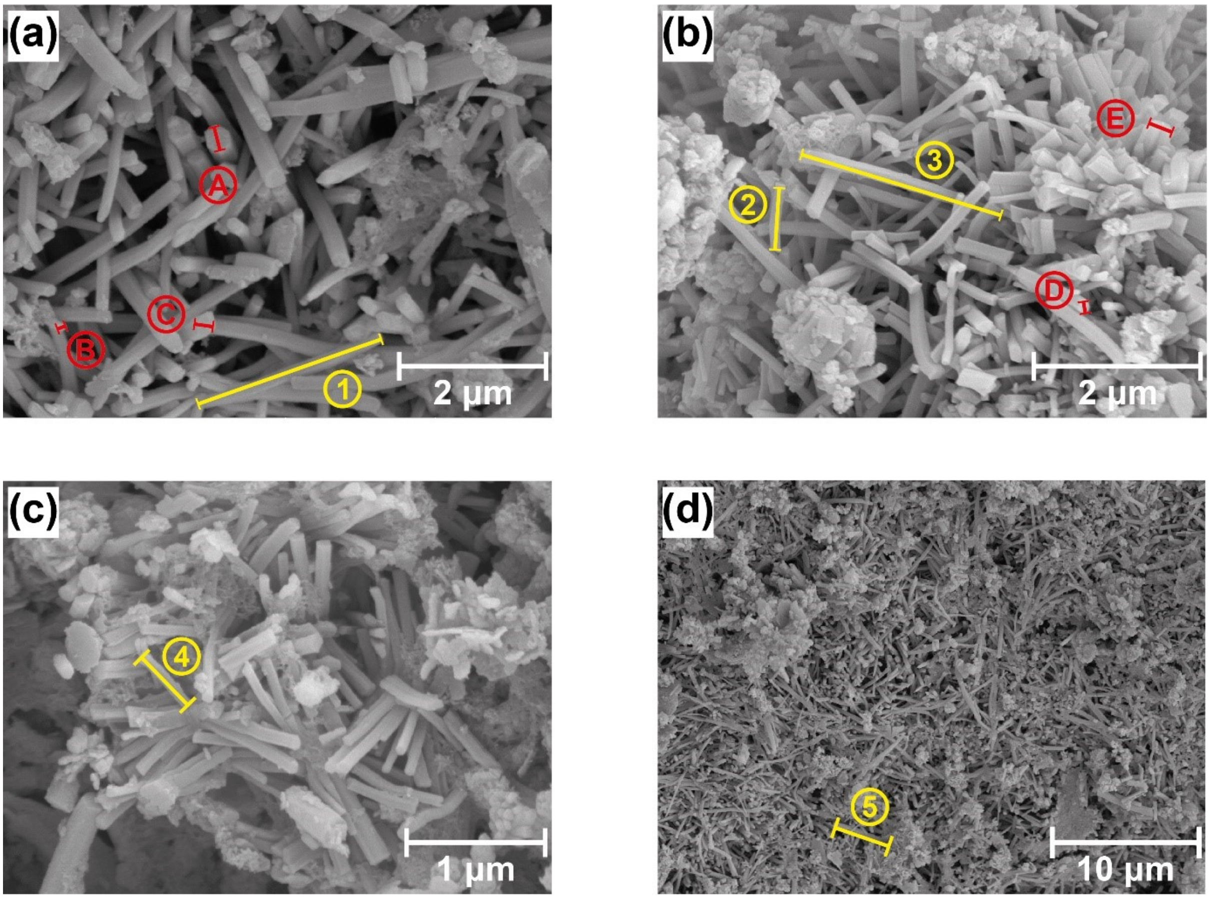

Figure S3: Selection of SEM images from a range of B-CuHHTP samples. The measurements shown (summarised in Table S3 below) were used to determine the length-to-aspect ratio of the 'rod-like' particles.

Table S3: Determination of Length-to-Width Aspect for B-CuHHTP from SEM Images

Label	Dimension	Measurement / µm
1	Length	2.60
2	Length	0.73
3	Length	2.26
4	Length	0.48
5	Length	4.03
A	Width	0.34
B	Width	0.11
C	Width	0.22
D	Width	0.17
E	Width	0.27

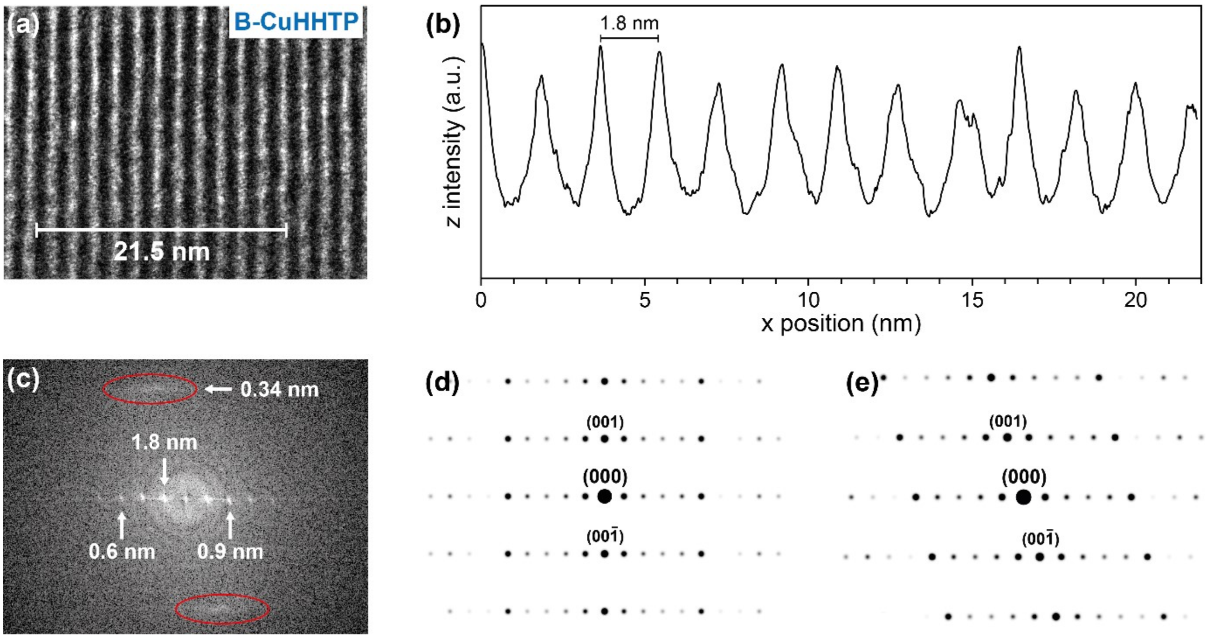

Figure S4: (a) TEM image of a 'rod-like' particle of B-CuHHTP, imaged parallel to the length of the particle. (b) Line intensity plot of the image, from which an inter-fringe spacing of 1.8 nm is determined. (c) Corresponding fast Fourier transform (FFT) image, confirming this value. Simulated FFT images from $Cu_3(HHTP)_2$ viewed along the *a* axis with (d) eclipsed stacking, and (e) near-eclipsed stacking. These simulations support XRD observations that B-CuHHTP has near-eclipsed layer stacking.

Table S4: Electrical Conductivity Measurement Data from Two-Point Measurement

Sample	Area of Pellet, A / cm^2	Thickness of Pellet, L / cm	Measured Resistance at 4 ton-force cm^{-2} / Ω	Conductivity, σ / S cm^{-1}
A-CuHHTP	1.33	0.0168 ± 0.0025	7.26	0.00174 ± 0.00026
B-CuHHTP	1.33	0.0153 ± 0.0015	5.29	0.00222 ± 0.00022
C-CuHHTP	1.33	0.0283 ±0.0020	10.5	0.00204 ± 0.00014

Table S5: Predicted & Experimental Elemental Analysis Results from $Cu_3(HHTP)_2$

Element	Predicted / wt%	A-CuHHTP / wt%	B-CuHHTP / wt%	C-CuHHTP / wt%	A-CuHHTP (long wash) / wt%
Cu	23.1	21.7	24.1	17.8	20.4
C	52.3	48.9	45.8	55.1	46.4
H	1.5	2.4	2.6	2.35	2.32
N	0	2.8	< 0.2	2.25	2.31

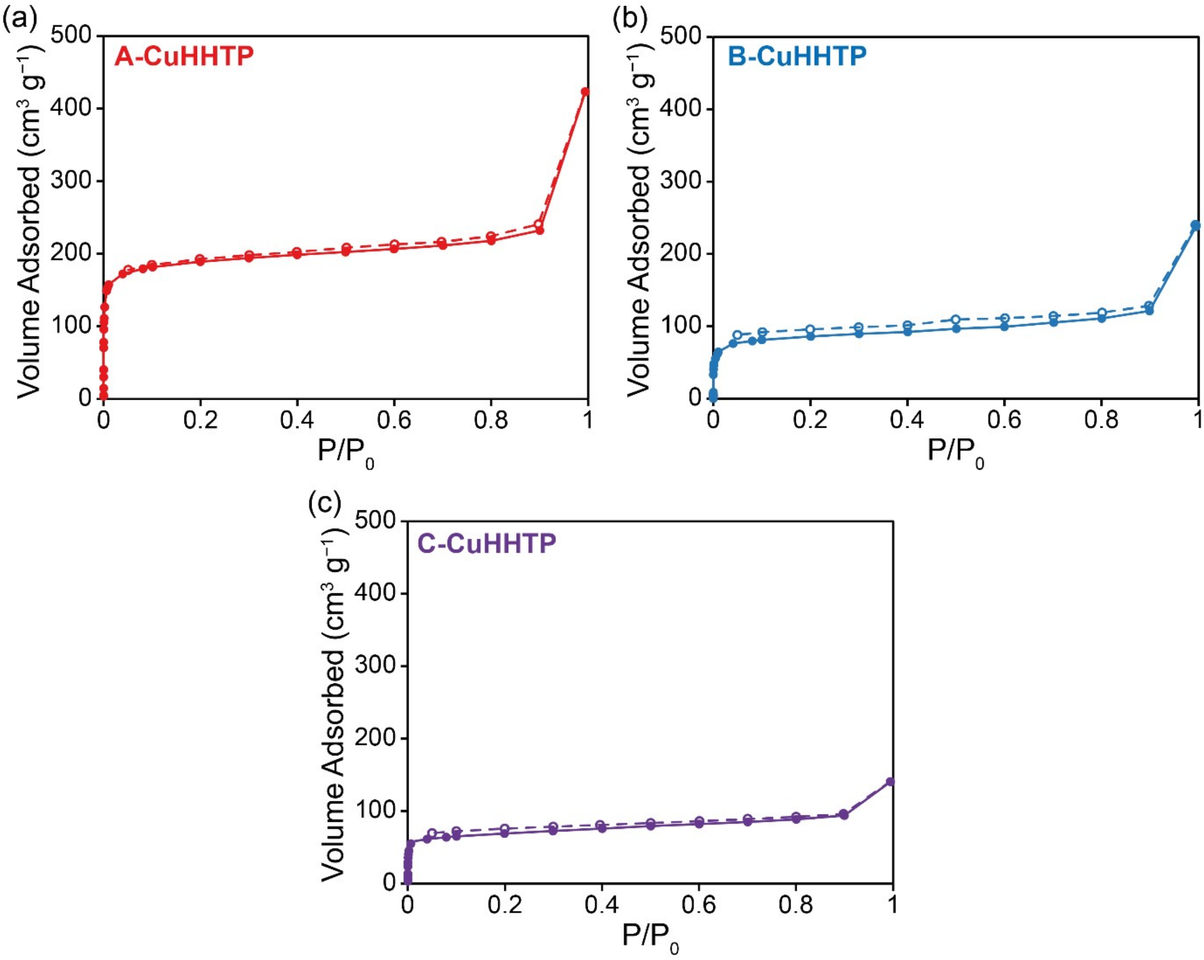

Figure S5: 77 K N_2 sorption isotherms from (a) A-CuHHTP, (b) B-CuHHTP, and (c) C-CuHHTP. Adsorption isotherms are shown with blocked lines and filled circles, and desorption isotherms are shown with dashed lines and unfilled circles.

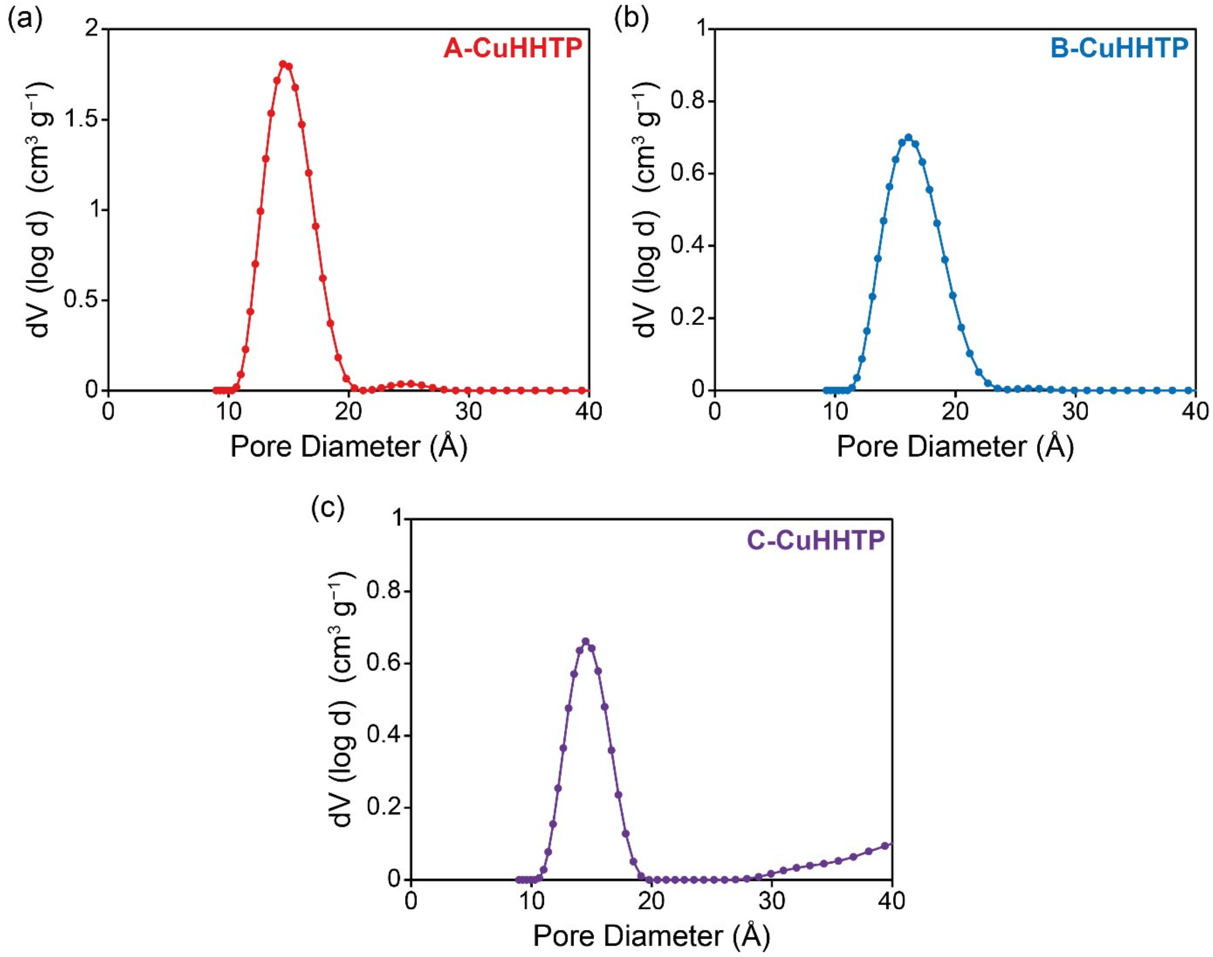

Figure S6: Pore size distributions of (a) A-CuHHTP, (b) B-CuHHTP, (c) C-CuHHTP determined by QSDFT modelling of the N_2 sorption isotherms from each sample with a cylindrical pore carbon model.

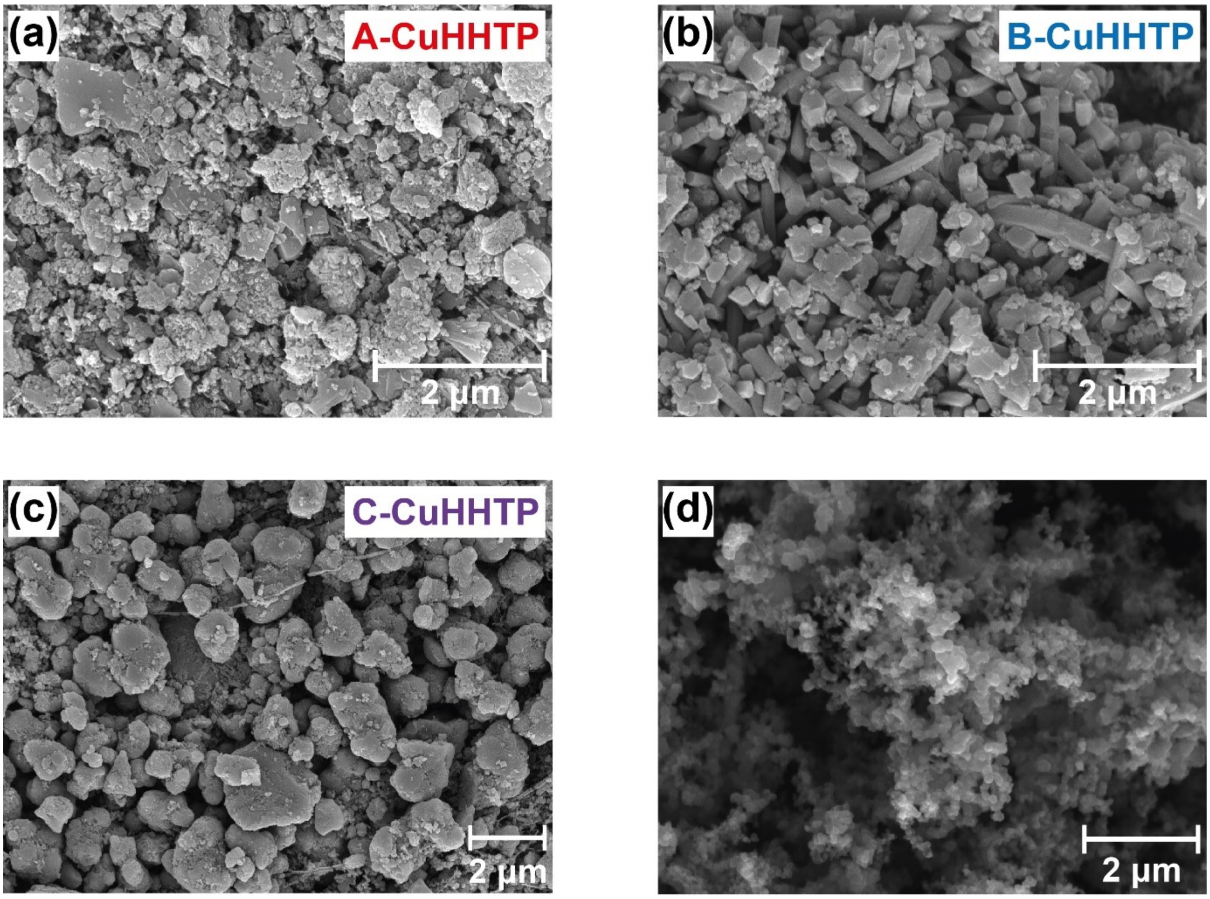

Figure S7: SEM images from composite films of (a) A-CuHHTP, (b) B-CuHHTP, (c) C-CuHHTP, showing that particle microstructures are largely maintained following film formation. Note that the small nanoscale particles seen in the images of these films are likely due to the acetylene black conductive additive (d).

Table S6: Physical Properties of Supercapacitor Cells Used in GCD Experiments

Sample	Cell	Electrolyte	Areal mass loading of $Cu_3(HHTP)_2$ per electrode / mg cm^{-2}	Internal Resistance / Ω (GCD, 0.05 A g^{-1})	Internal Resistance / Ω (EIS)
**A-CuHHTP**	**1**	**1 M NEt_4BF_4/ACN**	**16.4**	**8.1**	**4.5**
A-CuHHTP	2	1 M NEt_4BF_4/ACN	14.9	12.3	7.3
**B-CuHHTP**	**3**	**1 M NEt_4BF_4/ACN**	**15.4**	**8.6**	**5.9**
B-CuHHTP	4	1 M NEt_4BF_4/ACN	16.9	8.6	5.1
**C-CuHHTP**	**5**	**1 M NEt_4BF_4/ACN**	**14.6**	**3.9**	**2.5**
C-CuHHTP	6	1 M NEt_4BF_4/ACN	17.9	6.1	3.3
**A-CuHHTP**	**A**	**EMIM-BF_4**	**9.3**	**50.7**	**48.4**
A-CuHHTP	B	EMIM-BF_4	9.8	46.5	45.6
**B-CuHHTP**	**C**	**EMIM-BF_4**	**8.6**	**47.2**	**52.1**
B-CuHHTP	D	EMIM-BF_4	9.8	44.7	43.2
**C-CuHHTP**	**E**	**EMIM-BF_4**	**9.6**	**60.5**	**70.4**
C-CuHHTP	F	EMIM-BF_4	7.2	81.1	85.6

*The CV and EIS data shown in Figures 4 and 5 of the main text are from the cells highlighted in bold.

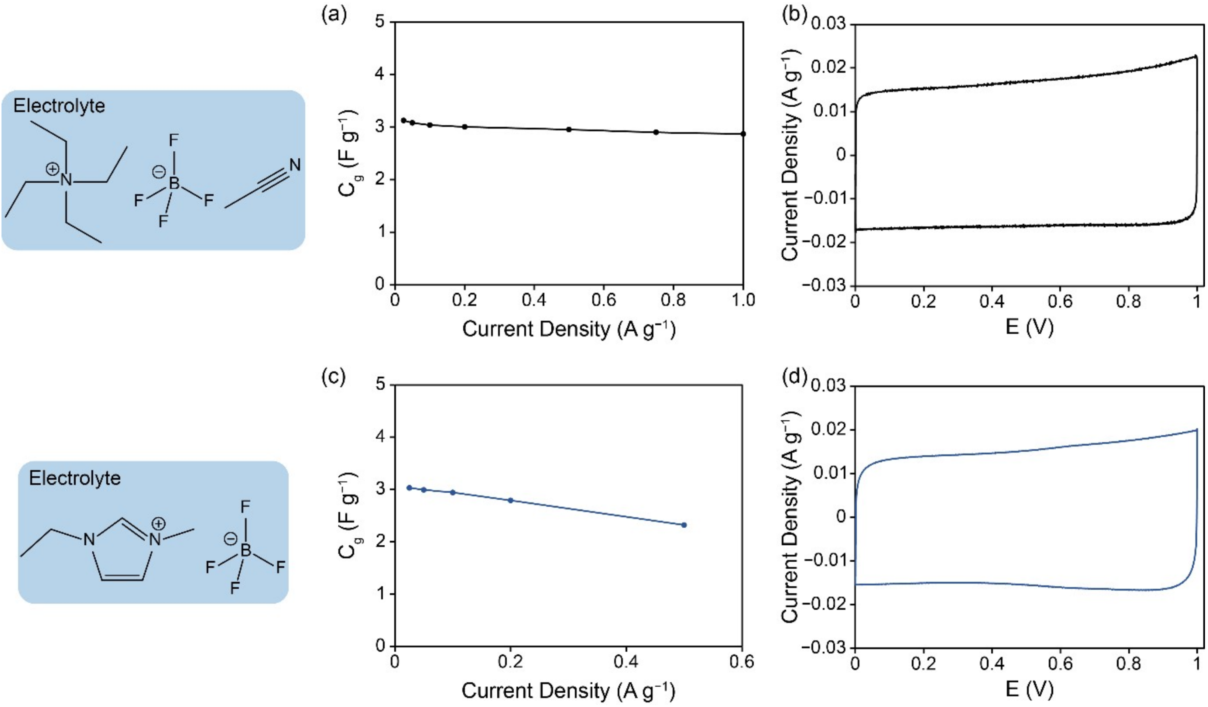

Figure S8: Electrochemical characterisation data from symmetric supercapacitors assembled with acetylene black electrodes and (a), (b) 1 M NEt_4BF_4/ACN electrolyte; (c), (d) EMIM-BF_4 electrolyte.

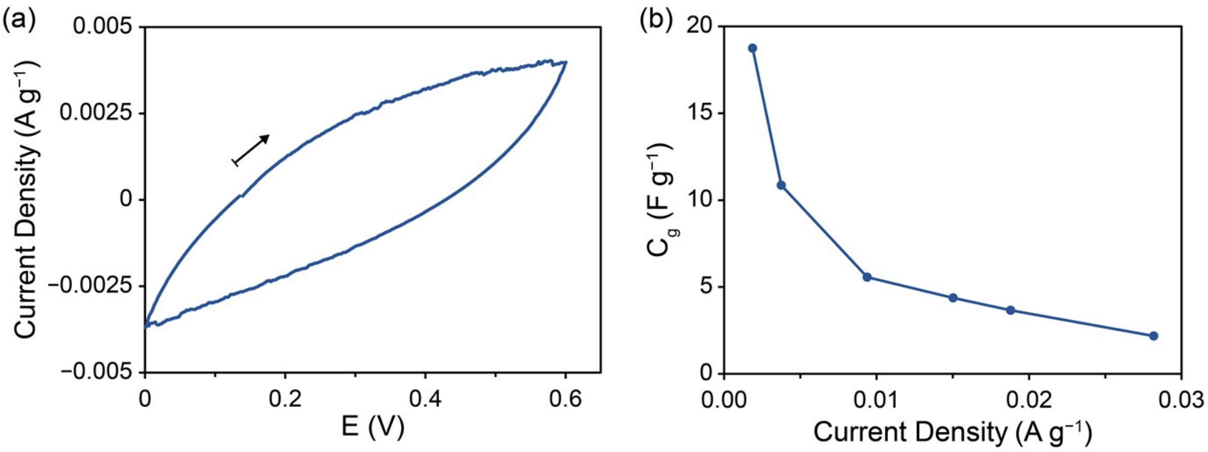

Figure S9: (a) CV obtained at a scan rate of 10 mV s^{-1}, and (b) specific capacitance versus current density curve obtained from GCD experiments with charging up to 0.6 V from a symmetric supercapacitor assembled with neat A-CuHHTP pellet electrodes and 1 M NEt_4BF_4/ACN electrolyte. This demonstrates the poor energy storage performances of neat $Cu_3(HHTP)_2$ pellets in supercapacitors.

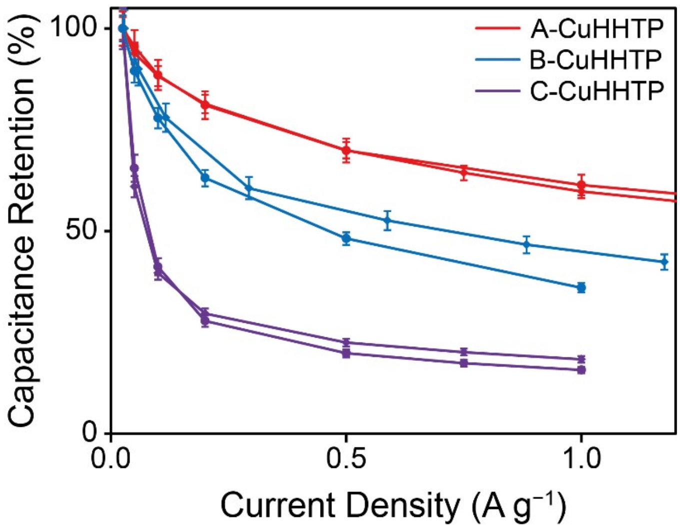

Figure S10: Comparison of capacitance retention versus current density plots from symmetric supercapacitors constructed with A-CuHHTP, B-CuHHTP and C-CuHHTP composite electrodes and 1 M NEt_4BF_4/ACN, showing the lower capacitance retention of B-CuHHTP and C-CuHHTP with this electrolyte. Data are normalised to the capacitance value at 0.025 A g^{-1}.

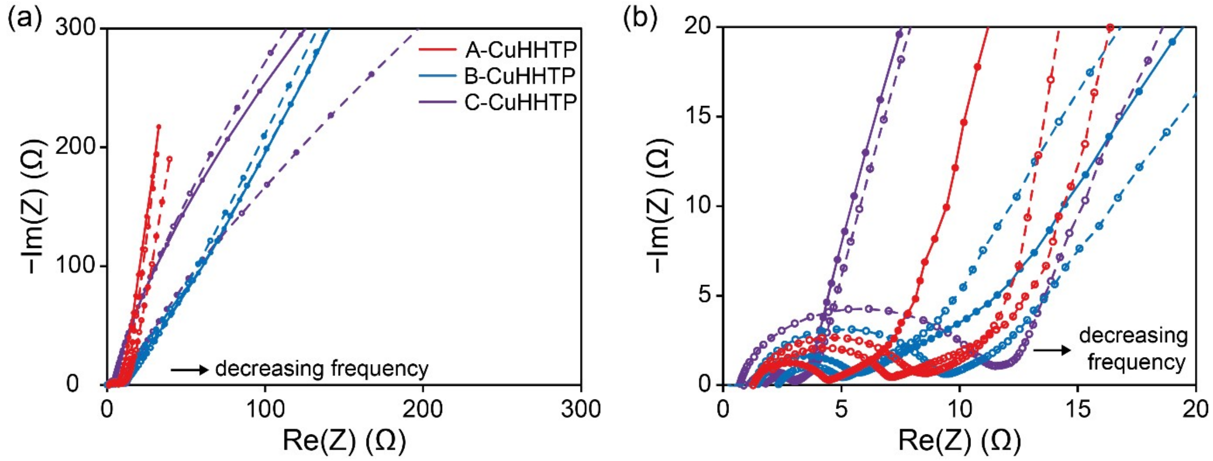

Figure S11: (a) Nyquist plots from EIS experiments on symmetric supercapacitors assembled with A-CuHHTP, B-CuHHTP, and C-CuHHTP composite electrodes and 1 M NEt_4BF_4/ACN, showing the reproducibility of the results. (b) Zoomed in view to show the high and intermediate frequency domains in greater detail.

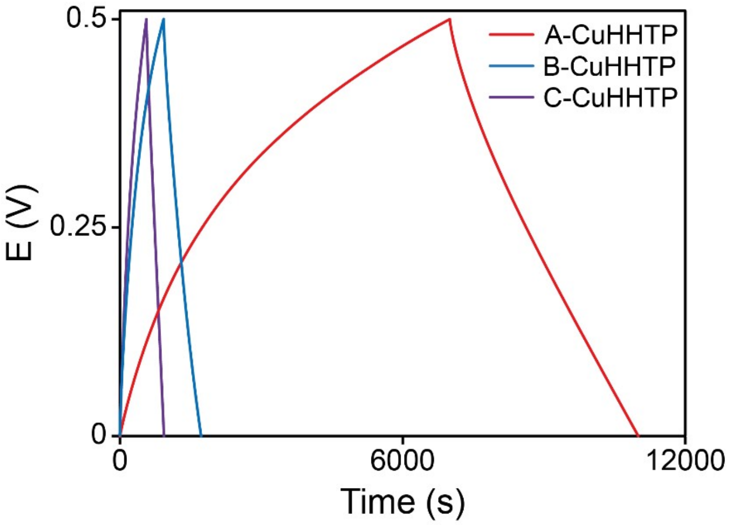

Figure S12: Galvanostatic charge-discharge (GCD) profiles at a current density of 0.0025 A g^{-1} from symmetric supercapacitors constructed with A-CuHHTP, B-CuHHTP and C-CuHHTP composite electrodes and EMIM-BF_4 ionic liquid. This demonstrates the lower charge storage in B-CuHHTP and C-CuHHTP with this electrolyte even at very low current densities.

Table S7: Slow Charging of Supercapacitors with EMIM-BF_4

Sample	Cell	Specific Capacitance, C_g (0.0025 A g^{-1}, 0.5 V*) / F g^{-1}
A-CuHHTP	A	46.5
B-CuHHTP	C	7.1
C-CuHHTP	E	2.1

*Charging was limited to 0.5 V to avoid kinetically-limited degradation that has been shown to occur in this system at *ca.* 0.8 V when charging very slowly.[1]

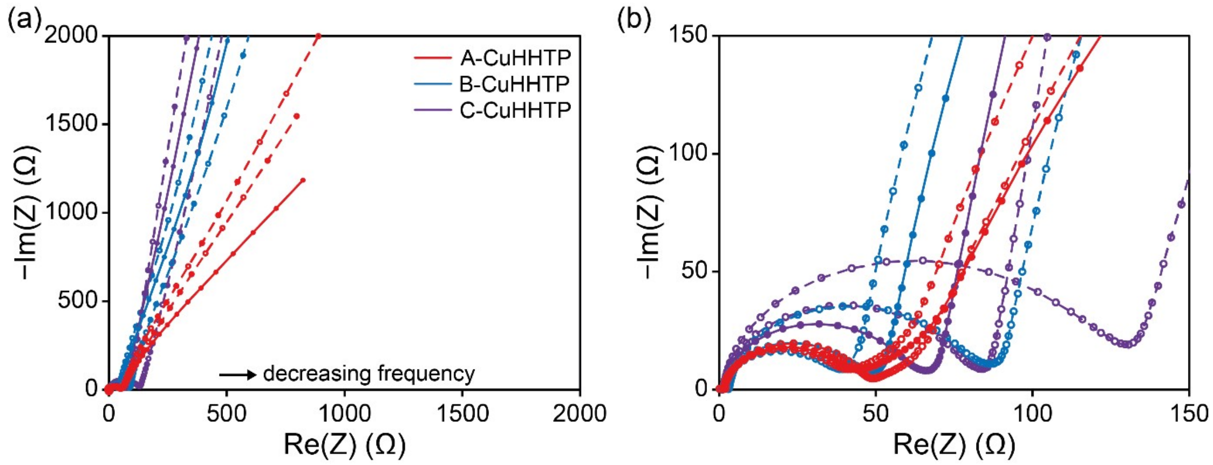

Figure S13: (a) Nyquist plots from EIS experiments on symmetric supercapacitors assembled with A-CuHHTP, B-CuHHTP, and C-CuHHTP composite electrodes and neat EMIM-BF_4 electrolyte, showing the reproducibility of the results. (b) Zoomed in view to show the high and intermediate frequency domains in greater detail.

Supplementary References

(1) Gittins, J. W.; Balhatchet, C. J.; Chen, Y.; Liu, C.; Madden, D. G.; Britto, S.; Golomb, M. J.; Walsh, A.; Fairen-Jimenez, D.; Dutton, S. E.; Forse, A. C. Insights into the Electric Double-Layer Capacitance of Two-Dimensional Electrically Conductive Metal–Organic Frameworks. *Journal of Materials Chemistry A* **2021**, *9* (29), 16006–16015. https://doi.org/10.1039/d1ta04026j.

(2) Day, R. W.; Bediako, D. K.; Rezaee, M.; Parent, L. R.; Skorupskii, G.; Arguilla, M. Q.; Hendon, C. H.; Stassen, I.; Gianneschi, N. C.; Kim, P.; Dincă, M. Single Crystals of Electrically Conductive Two-Dimensional Metal-Organic Frameworks: Structural and Electrical Transport Properties. *ACS Central Science* **2019**, *5* (12), 1959–1964. https://doi.org/10.1021/acscentsci.9b01006.

(3) Momma, K.; Izumi, F. VESTA 3 for Three-Dimensional Visualization of Crystal, Volumetric and Morphology Data. *Journal of Applied Crystallography* **2011**, *44* (6), 1272–1276. https://doi.org/10.1107/S0021889811038970.

(4) Schneider, C. A.; Rasband, W. S.; Eliceiri, K. W. NIH Image to ImageJ: 25 Years of Image Analysis. *Nature Methods* **2012**, *9* (7), 671–675. https://doi.org/10.1038/nmeth.2089.

(5) Gómez-Gualdrón, D. A.; Moghadam, P. Z.; Hupp, J. T.; Farha, O. K.; Snurr, R. Q. Application of Consistency Criteria to Calculate BET Areas of Micro- and Mesoporous Metal-Organic Frameworks. *Journal of the American Chemical Society* **2016**, *138* (1), 215–224. https://doi.org/10.1021/jacs.5b10266.

(6) Brunauer, S.; Emmett, P. H.; Teller, E. Adsorption of Gases in Multimolecular Layers. *Journal of the American Chemical Society* **1938**, *60* (2), 309–319. https://doi.org/10.1021/JA01269A023.

(7) Thommes, M.; Kaneko, K.; Neimark, A. v.; Olivier, J. P.; Rodriguez-Reinoso, F.; Rouquerol, J.; Sing, K. S. W. Physisorption of Gases, with Special Reference to the Evaluation of Surface Area and Pore Size Distribution (IUPAC Technical Report). *Pure and Applied Chemistry* **2015**, *87* (9–10), 1051–1069. https://doi.org/ 10.1515/pac-2014-1117.

(8) Mei, B.-A.; Munteshari, O.; Lau, J.; Dunn, B.; Pilon, L. Physical Interpretations of Nyquist Plots for EDLC Electrodes and Devices. *Journal of Physical Chemistry C* **2018**, *122* (1), 194–206. https://doi.org/10.1021/acs.jpcc.7b10582.
